# Network analysis of psoriasis reveals biological pathways and roles for coding and long non-coding RNAs

**DOI:** 10.1186/s12864-016-3188-y

**Published:** 2016-10-28

**Authors:** Richard Ahn, Rashmi Gupta, Kevin Lai, Nitin Chopra, Sarah T. Arron, Wilson Liao

**Affiliations:** 1 Department of Dermatology, University of California, San Francisco, 2340 Sutter Street, Box 0808, San Francisco, CA 94143-0808 USA; 2 Current address: Icahn School of Medicine at Mount Sinai, New York, NY USA

**Keywords:** Psoriasis, Gene expression, RNA-seq, Long non-coding RNA (lncRNA), Weighted gene coexpression network analysis (WGCNA)

## Abstract

**Background:**

Psoriasis is an immune-mediated, inflammatory disorder of the skin characterized by chronic inflammation and hyperproliferation of the epidermis. Differential expression analysis of microarray or RNA-seq data have shown that thousands of coding and non-coding genes are differentially expressed between psoriatic and healthy control skin. However, differential expression analysis may fail to detect perturbations in gene coexpression networks. Sensitive detection of such networks may provide additional insight into important disease-associated pathways. In this study, we applied weighted gene coexpression network analysis (WGCNA) on RNA-seq data from psoriasis patients and healthy controls.

**Results:**

RNA-seq was performed on skin samples from 18 psoriasis patients (pre-treatment and post-treatment with the TNF-α inhibitor adalimumab) and 16 healthy controls, generating an average of 52.3 million 100-bp paired-end reads per sample. Using WGCNA, we identified 3 network modules that were significantly correlated with psoriasis and 6 network modules significantly correlated with biologic treatment, with only 16 % of the psoriasis-associated and 5 % of the treatment-associated coexpressed genes being identified by differential expression analysis. In a majority of these correlated modules, more than 50 % of coexpressed genes were long non-coding RNAs (lncRNA). Enrichment analysis of these correlated modules revealed that short-chain fatty acid metabolism and olfactory signaling are amongst the top pathways enriched for in modules associated with psoriasis, while regulation of leukocyte mediated cytotoxicity and regulation of cell killing are amongst the top pathways enriched for in modules associated with biologic treatment. A putative autoantigen, LL37, was coexpressed in the module most correlated with psoriasis.

**Conclusions:**

This study has identified several networks of coding and non-coding genes associated with psoriasis and biologic drug treatment, including networks enriched for short-chain fatty acid metabolism and olfactory receptor activity, pathways that were not previously identified through differential expression analysis and may be dysregulated in psoriatic skin. As these networks are comprised mostly of non-coding genes, it is likely that non-coding genes play critical roles in the regulation of pathways involved in the pathogenesis of psoriasis.

**Electronic supplementary material:**

The online version of this article (doi:10.1186/s12864-016-3188-y) contains supplementary material, which is available to authorized users.

## Background

Psoriasis is an immune-mediated, inflammatory disorder of the skin, is characterized by chronic inflammation and subsequent hyper proliferation of the epidermis that results in silvery scales and a thickening of the skin. In the past decade, microarray-based differential expression studies have shown that hundreds of genes that are differentially expressed between psoriatic and healthy control skin [1–4]. More recently, RNA-seq-based differential expression studies, including a study by Li et al. [5], have dramatically increased the number of differentially expressed genes (DEGs) found between psoriatic and healthy skin, with the number of known DEGs in the thousands.

However, while differential expression analyses have successfully revealed transcriptomic signatures comprised of many individual DEGs, differential expression analysis may fail to detect important biological pathways or gene-gene interactions associated with disease due to a focus on the effect of individual genes rather than on the effect of networks of genes. Gene coexpression network analysis methods were developed to understand the relationship between pairs of genes and ultimately, gene networks or modules that are associated with a distinct biological function. Unweighted gene coexpression methods constructed these networks using pairwise correlations [6], Bayesian graphical models [7], or linear regression [8]. Weighted gene coexpression network analysis (WGCNA) [9] builds upon these previous unweighted methods by implementing a correlation-based soft-thresholding weight that prioritizes the strongest pairwise correlations and penalizes weaker ones and complements differential expression analysis by testing for association between a disease and networks of correlated genes. Unlike methods such as Gene Set Enrichment Analysis [10], the WGCNA framework is based on the rationale that gene networks can be constructed with gene correlation matrices alone, without prior network or pathway information that can introduce bias. Furthermore, WGCNA offers a way to prioritize the most important genes in a given network by calculating a measure of connectivity for each gene which is based on the number of correlations between each gene and all other genes in the network. A WGCNA-based screen reached a higher validation rate than a differential expression analysis based approach in identifying a biomarker for glioblastoma [11]. WGCNA has also been successfully applied in screening for disease-associated pathways, molecular targets, and candidate genes in chronic fatigue syndrome [12], Sjögren’s Syndrome [13], coronary heart disease [14], and inflammatory bowel disease [15].

Although WGCNA has been used to identify networks of coding genes associated with psoriasis [5, 16], WGCNA has not been used to identify networks of coding genes and long non-coding RNAs (lncRNAs). Therefore, we applied WGCNA to coding genes and lncRNAs sequenced by RNA-seq on lesional skin samples from psoriasis patients before (PP) and after treatment (PT) with a TNF-α inhibitor, adalimumab, and on healthy control skin (NN). Here we report the identification of novel networks of coding genes and lncRNAs associated with psoriasis and response to therapy and show that WGCNA uncovers additional biological pathways compared to differential expression analysis alone.

## Methods

### RNA-seq and differential expression of coding genes and lncRNAs in lesional psoriatic skin tissue

18 adult subjects with chronic plaque psoriasis were recruited from the University of California San Francisco (UCSF) Dermatology Department. A board certified dermatologist confirmed the diagnosis of psoriasis. The participants were required to have affected body surface area > 10 % and to not already be on systemic medications for their psoriasis. Among the 18 subjects, 4 were female, the mean age was 39.2 years (s.d. = 9.7 years), and the mean body mass index was 28.9 (s.d. = 6.2). Five millimeter punch biopsies were taken from the edge of a psoriatic plaque of each patient. Two skin biopsies were taken from each participant, the first prior to the initiation of adalimumab and the second one month after starting treatment. The mean Psoriasis Area and Severity Index (PASI) score prior to treatment was 14.6 (s.d. = 3.6) and after one month of adalimumab treatment was 7.0 (s.d. = 3.9), with a mean improvement of the PASI of 53.1 %. Sixteen normal skin samples were obtained from healthy control surgical discard specimens.

We prepared cDNA libraries from ribosome-depleted RNA extracted from skin biopsies of 18 psoriatic patients and 16 healthy controls that were sequenced on the Illumina HiSeq 2000 platform, which yielded an average of 52.3 million 100-bp paired-end reads per sample. Sequenced reads were aligned to the hg19/GRCh37 human reference genome using TopHat2 [17]. Gene annotations for 23 K coding genes and 67 K lncRNAs (including lincRNAs) were obtained from RefSeq [18] and Hangauer et al.’s dataset S3 [19], respectively. We used CuffDiff [20] to test for differential expression.

### Weighted gene coexpression network analysis

After expression values were normalized to the number of reads per kilobase per million reads, QC was performed on the matrix of normalized expression values to remove any transcripts with either zero variance or a missing value and remove samples that were outliers in an initial unsupervised hierarchical clustering analysis (9303 genes, 3 control samples). After QC, a weighted adjacency matrix was created, defined as, *A*_*ij*_ *= (|cor* ^*^ *(x*_*i,*_ *x*_*j*_ *)|* ^*β*^, where *x*_*i*_ and *x*_*j*_ are the *i-th* and *j-th* genes, respectively. The soft thresholding power parameter, *β*, was set to 6 after a sensitivity analysis of scale-free topology was performed. This weighted adjacency matrix was used to generate a topological overlap matrix (TOM) and dendrogram. A dynamic hybrid branch cutting method was implemented on the resulting TOM-based dendrogram to identify module eigengenes (ME). MEs are the first principal components for each gene expression module after a singular value decomposition is performed on the TOM. A cut height of 0.2 was set to merge MEs that have a correlation of 0.8 or greater. A phenotypic trait-based gene significance measure was defined as *GS*_*i*_ *= −*log_10_(p_|cor*(xi,t)|_), where *x*_*i*_ is the *i-th* gene and *t* is the binary indicator variable for psoriasis case status (or treatment status). An ME significance was defined as *MES*_*i*_ *= |cor* ^*^ *(ME*_*j*_ *,t)|*, where *ME*_*j*_ is the *j-th* ME. Module membership, MM, for the *i-th* gene was defined as, MM *= |cor* ^*^ *(x*_*i*_ *,ME)|.*

^*^Unless otherwise specified, ‘cor’ refers to Pearson correlation.

### Pathway analysis

We uploaded lists of DEGs and lists of genes from the significantly correlated modules between NN and PP and between PP and PT to the Illumina NextBio Research platform for GO term enrichments and Broad MSigDB Canonical Pathways enrichment. DAVID (https://david.ncifcrf.gov) was used to find KEGG pathway enrichments.

## Results

### Network analysis of coding genes and lncRNAs in healthy and psoriatic skin

We analyzed RNA-seq data from 18 psoriatic patients and 16 healthy controls previously described in Gupta et al. [21]. Traditional differential expression analysis in PPvNN revealed that 5328 genes were differentially expressed (FDR ≤ 0.05), including 4357 coding genes and 971 lncRNAs (Additional file 1). In PPvPT, 2657 genes were differentially expressed (FDR ≤ 0.05), including 2500 coding genes and 157 lncRNAs (Additional file 1). A validation of the DE lncRNAs was performed by implementing reverse transcriptase qPCR on lncRNAs from 17 cases and 14 healthy controls. Four DE lncRNAs (*TRHDE-AS1*, *CYP4Z2P*, *HINT1*, and *RPSAP58*) were chosen for validation, with three of the four lncRNAs (*TRHDE-AS1*, *CYP4Z2P*, and *HINT1*) being DE in the same direction as found in the RNA-seq analysis. A detailed description of differential expression analysis of lncRNAs and validation of DE lncRNAs by qPCR is provided in Gupta et al. [21]. The top 3 GO terms from PPvNN were viral reproductive process (*p* = 1.40e-98), nuclear division (*p* = 1.30e-87), and mitosis (*p* = 1.30e-87), all with significantly up-regulated genes. From PPvPT, the top 3 GO terms were mitosis (*p* = 5.60e-100), nuclear division (*p* = 5.60e-100), and viral reproductive process (*p* = 6.80e-93), all with significantly down-regulated genes. The full list of GO terms are in Additional file 2.

To determine if a network analysis approach reveals psoriasis-associated biological pathways that were not previously found by differential expression analysis, we started by asking which coding and non-coding genes are uniquely identified by network analysis versus differential expression analysis. To answer this question, we implemented WGCNA to identify module eigengenes (MEs) that correlated with either psoriasis or with positive response to biologic treatment. MEs are the ideal unit to correlate with external traits because they are the first principal component of a network of coexpressed genes and thus account for the most variance in the data. We generated 64 MEs in PPvNN, 3 of which were significantly correlated with psoriasis (7849 genes; FDR ≤ 0.05) (Table 1). In PPvPT, we generated 70 MEs, 6 of which were significantly correlated with positive response to biologic treatment (5775 genes; FDR ≤ 0.05) (Table 2).

**Table 1:** Top module eigengenes that are significantly correlated with psoriasis (FDR ≤ 0.05) between PP and NN

Module	Trait Correlation	FDR_Trait Correlation_	# of genes	# of lncRNA	% lncRNA	# DE RefSeq	# DE lncRNAs
blue	−0.86	2.42E-08	5719	2655	46 %	1031	236
salmon	0.81	1.39E-06	2002	1820	91 %	3	0
lavenderblush3	0.74	3.36E-05	128	113	88 %	0	0

**Table 2:** Top module eigengenes that are significantly correlated with psoriasis (FDR ≤ 0.05) between PP and PT

Module	Trait Correlation	FDR_Trait Correlation_	# of genes	# of lncRNA	% lncRNA	# DE RefSeq	#DE lncRNA
sienna3	0.71	8.32E-05	564	470	83 %	1	0
lightyellow	−0.66	4.28E-04	969	792	82 %	1	0
salmon	−0.6	2.91E-03	1290	459	36 %	169	5
black	0.53	1.48E-02	2339	1813	78 %	27	1
coral1	−0.5	2.92E-02	130	123	95 %	0	0
mediumpurple3	−0.48	3.19E-02	483	168	35 %	98	1

**Fig. 1:**
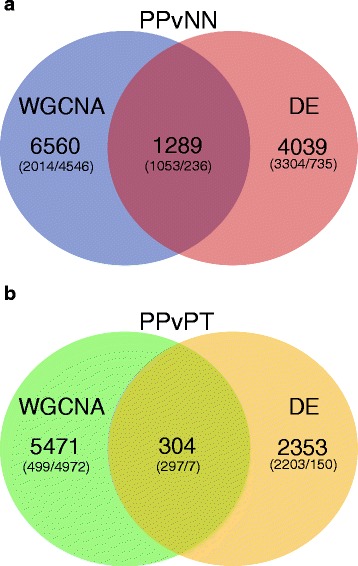
WGCNA identifies genes associated with psoriasis **a** and biologic treatment **b** not identified by DE. Venn diagram of genes identified by WGCNA or DE that are associated with psoriasis in PPvNN **a** or with biologic treatment in PPvPT **b**. Values in parantheses are the count of coding genes to lncRNAs

Although there was some overlap between genes identified by DE and WGCNA, the WGCNA approach identified a large number of genes significantly correlated with psoriasis and biologic treatment that were not identified by differential expression analysis (Fig. 1). We found that 84 % and 95 % of genes identified by WGCNA as being associated with psoriasis (PPvNN) or psoriasis treatment (PPvPT), respectively, were not identified by DE analysis. In PPvNN, 93 coding genes that were exclusively identified via DE were amongst the top 100 over-expressed coding genes in PP while 22 coding genes identified via DE only were amongst the top 100 under-expressed coding genes in PP. 87 lncRNAs identified via DE only were amongst the top 100 over-expressed lncRNAs while 52 such lncRNAs were amongst the top 100 under-expressed lncRNAs. The 93 over-expressed coding genes were significantly enriched for GO terms such as keratinization (*p* = 3.69e-20), keratinocyte differentiation (*p* = 1.82e-15), and cytokine-mediated signaling pathway (*p* = 1.18e-8), replicating results from previous studies.

For PPvPT, 73 coding genes identified via DE only were amongst the top 100 over-expressed coding genes in PT, while 99 coding genes were amongst the top 100 under-expressed coding genes in PT. Of the 157 DE lncRNAs in PPvPT, 150 were exclusively identified by DE analysis, with 9 lncRNAs under-expressed and 141 lncRNAs over-expressed in PP. Unsurprisingly, the 99 under-expressed coding genes were significantly enriched for keratinization (*p* = 4.43e-9), keratinocyte differentiation (*p* = 1.41e-7), and inflammatory response (*p* = 2.82e-7), which coincides with the efficacy of the treatment with adalimumab.

We next examined the two most significantly correlated modules from both PPvNN and PPvPT. For PPvNN, the module most significantly correlated with psoriasis was the blue module (ρ = −0.86, *p* = 3.77e-10). The negative correlation indicates that the genes in this module were underexpressed in PP. The top 3 GO terms that were significantly enriched for in the blue module included “lipid biosynthetic process” (*p* = 2.50e-61), “fatty acid metabolic process” (*p* = 4.10e-57), and “mitochondrial matrix” (*p* = 3.90e-53) (Table 3; Additional file 3). With 5719 genes, the blue module was the largest significant module and also had the greatest proportion of differentially expressed genes (DEG). 54 % of the blue module genes were coding genes and 34 % of these genes were DEGs. Of the 46 % of blue module genes that were lncRNAs, 9 % were DEGs. None of the top blue module GO terms were amongst the top 20 GO terms enriched for in the DE genes.

**Table 3:** Top 10 GO Term and Broad MSigDB Canonical Pathway enrichments for 2 most correlated modules in PPvNN

Module	GO Term	p_GO_	Broad MSigDB Canonical Pathway	p_Broad_
Blue	lipid biosynthetic process	2.50E-61	Genes involved in Metabolism of lipids and lipoproteins	6.10E-71
	fatty acid metabolic process	4.10E-57	Peroxisome	5.30E-39
	mitochondrial matrix	3.90E-53	Valine, leucine and isoleucine degradation	5.60E-35
	mitochondrial membrane	3.00E-52	Genes involved in Generic Transcription Pathway	6.90E-34
	mitochondrial inner membrane	2.20E-51	Genes involved in Fatty acid, triacylglycerol, and ketone body metabolism	5.90E-32
	organic acid catabolic process	1.70E-48	Genes involved in Cholesterol biosynthesis	2.90E-26
	peroxisome	5.30E-42	Genes involved in The citric acid (TCA) cycle and respiratory electron transport	6.40E-23
	microbody	5.30E-42	Fatty acid metabolism	1.20E-20
	cofactor binding	5.40E-40	Propanoate metabolism	2.40E-20
	steroid metabolic process	3.00E-36	Genes involved in Phospholipid metabolism	2.60E-18
Salmon	immune effector process	0.0001	Olfactory transduction	3.20E-16
	innate immune response	0.0002	Genes involved in Olfactory Signaling Pathway	2.70E-12
	cell surface	0.0002	Genes involved in Hyaluronan uptake and degradation	5.90E-05
	lymphocyte mediated immunity	0.0002	Genes involved in Hyaluronan metabolism	0.0001
	adaptive immune response based on somatic recombination of immune receptors built from immunoglobulin superfamily domains	0.0003	Genes involved in Glycosaminoglycan metabolism	0.0003
	adaptive immune response	0.0004	Sulfur metabolism	0.0006
	leukocyte mediated immunity	0.0004	Genes involved in Chondroitin sulfate biosynthesis	0.0009
	ion channel activity	0.0005	Glycosaminoglycan biosynthesis - chondroitin sulfate	0.001
	positive regulation of immune response	0.0005	PAR1-mediated thrombin signaling events	0.001
	response to bacterium	0.0006	Genes involved in Developmental Biology	0.0013

The next most significantly psoriasis-correlated module was the salmon module (ρ = 0.81, *p* = 4.33e-8). The positive correlation indicates that the genes in this module were overexpressed in PP. The top 3 GO terms that were significantly enriched for are “immune effector process” (*p* = 1.0e-4), “innate immune response” (*p* = 2.0e-4), and “cell surface” (*p* = 2.0e-4) as well as the Broad MSigDB canonical pathways, “olfactory transduction” (*p* = 3.20e-16) and “genes involved in olfactory signaling pathway” (*p* = 2.7e-12) (Table 3; Additional file 3). In contrast to the blue module, 90 % of the 2002 genes in the salmon module were lncRNAs and of the 182 coding genes, only 3 were DEGs, with none of the lncRNAs being DEGs. With the exception of “innate immune response”, none of the top salmon module GO terms were amongst the top 20 terms enriched for in the DE genes.

For PPvPT, the module most correlated with treatment response was the sienna3 module (ρ = 0.71, *p* = 1.18e-6). The top 3 GO terms that were enriched for are “regulation of leukocyte mediated cytotoxicity” (*p* = 1.40e-5), “regulation of cell killing” (*p* = 1.90e-5), and “leukocyte activation” (*p* = 1.0e-4) (Table 4; Additional file 4). A majority of the 564 genes (83 %) in the sienna3 module were lncRNAs. Almost none of the genes were differentially expressed, with just one coding DEG.

**Table 4:** Top 10 GO Term and Broad MSigDB Canonical Pathway enrichments for 2 most correlated modules in PPvPT

Module	GO Term	p_GO_	Broad MSigDB Canonical Pathway	p_Broad_
Sienna3	regulation of leukocyte mediated cytotoxicity	1.40E-05	Genes involved in Lipid digestion, mobilization, and transport	1.70E-05
	regulation of cell killing	1.90E-05	Antigen processing and presentation	0.0002
	leukocyte activation	0.0001	Genes involved in Neuronal System	0.0004
	T cell activation	0.0001	Genes involved in Lipoprotein metabolism	0.0004
	monosaccharide metabolic process	0.0001	Genes involved in Glucose metabolism	0.0004
	positive regulation of immune response	0.0002	Visual signal transduction: Cones	0.0005
	cytokine production	0.0003	Signaling events mediated by HDAC Class II	0.0008
	cell surface	0.0005	ABC transporters	0.0011
	MHC protein binding	0.0005	Downstream signaling in CD8+ T cells	0.0015
	microtubule organizing center	0.0006	Genes involved in Transmission across Chemical Synapses	0.0015
Lightyellow	protein complex disassembly	1.00E-10	Ribosome, cytoplasmic	2.10E-11
	protein targeting to ER	1.10E-10	Ribosome	3.50E-11
	establishment of protein localization to endoplasmic reticulum	1.10E-10	Genes involved in 3' -UTR-mediated translational regulation	6.90E-11
	viral infectious cycle	2.20E-10	Genes involved in Translation	3.70E-10
	macromolecular complex disassembly	3.10E-10	Genes involved in Peptide chain elongation	7.30E-10
	ribosomal subunit	4.50E-10	Genes involved in Influenza Viral RNA Transcription and Replication	1.40E-09
	cytosolic part	3.50E-09	Genes involved in Nonsense Mediated Decay Enhanced by the Exon Junction Complex	1.70E-09
	establishment of protein localization to organelle	5.70E-09	Genes involved in SRP-dependent cotranslational protein targeting to membrane	1.90E-09
	cellular component disassembly at cellular level	1.20E-08	Genes involved in Influenza Life Cycle	4.30E-09
	cellular component disassembly	1.30E-08	Genes involved in Metabolism of proteins	5.30E-09

The next most correlated module for treatment response in PPvPT was the lightyellow module (ρ = 0.66, *p* = 1.22e-5). GO terms that the lightyellow module was enriched for included “protein complex disassembly” (*p* = 1.0e-10), “protein targeting to ER” (*p* = 1.1e-10), and “establishment of protein localization to endoplasmic reticulum” (*p* = 1.1e-10) (Table 4; Additional file 4). Again the majority of the genes in this module were lncRNAs (82 %) and only one gene (coding) was a DEG. Interestingly, while nearly all of the overlapping DEGs from PPvNN (Fig. 1a) are found in the blue module, most of the 304 overlapping genes (Fig. 1b) from PPvPT are not found in the top three PPvPT modules. None of the top GO terms enriched for in either of the PPvPT sienna3 or lightyellow modules, were amongst the top 20 GO terms enriched for in the DE genes.

**Fig. 2:**
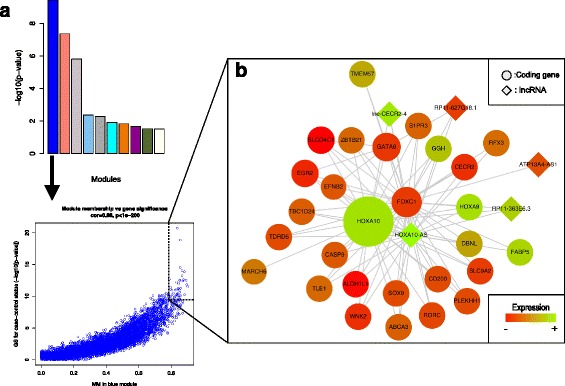
Intramodular analysis reveals hub genes in the top correlated module in PPvNN. Graphical illustration of intramodular analysis starting with identification of the most correlated modules and plotting MM against GS for the top correlated modules **a**. After hub genes are identified, a network plot of these genes is produced **b**. In this case, the network plot is of the hub genes of the PPvNN blue module. The relative size of each hub indicates the degree of connectivity (number of edges) for each gene

Next, we performed intramodular analysis to determine “hub genes” or genes that were the most connected to other genes and individually significant genes. Figure 2a graphically illustrates the process from identification of the most significant MEs to intramodular analysis. We defined a hub gene as a gene with gene significance (GS) ≥ 10 and module membership (MM) ≥ 0.8. We performed intramodular analysis on the top 3 modules in both PPvNN and PPvPT. Within the blue module of PPvNN, we identified 33 hub genes (Fig. 2b; Additional files 5), including *HOXA9*, *HOXA10*, and *GGH*. 28 of the 33 hub genes were lncRNAs and all but one of the hub genes (*MARCH6*) was differentially expressed in PPvNN. Genes in the other modules (for both PPvNN and PPvPT) did not meet GS or MM thresholds defined above (Additional files 6).

## Discussion

Differential expression analysis can identify individual genes that are differentially expressed between cases and controls. In this study, we investigated whether WGCNA would uncover biological pathways associated with psoriasis and treatment with a biologic drug that are not identified by differential expression analysis. Our results show that most of the genes identified in psoriasis or treatment-associated co-expression networks are not differentially expressed. We also inferred the function of non-coding genes that were coexpressed with coding genes in networks of genes correlated with psoriasis or treatment response by testing these coexpression networks for GO or KEGG term enrichment.

### Dominance of lncRNAs in network modules

We were surprised to discover that most of the genes in the majority of the modules that were significantly correlated with psoriasis were lncRNAs, particularly in PPvNN, where 2 of the 3 significantly psoriasis-correlated modules were at least 80 % lncRNA, with most of these lncRNAs not being DE. This dominance of non-DE lncRNAs in psoriasis-correlated modules may be due to the overall low-abundance of lncRNAs and inability of differential expression methods to detect minute (and statistically insignificant) but biologically significant differences in lncRNA expression, with coding genes having nearly ten times more abudance than lncRNAs on average [22]. As coexpression network analysis is based on pairwise correlations between genes and not on relative differences in expression between biological states, coexpression network analysis may be more robust to inclusion of low-abundance transcripts compared to differential expression analysis. This dominance of lncRNAs in psoriasis-correlated modules suggests the possibility that lncRNAs play a significant role in psoriasis pathogenesis through regulation of coding genes in key pathways. Previous studies of lncRNA indicate they can act by guiding chromatin modifiers and histone modifiers to targeted loci to regulate transcription, function as ligands for proteins, and play crucial roles in cell differentiation that ultimately determine cell fate [23].

### Module enriched for metabolic activity

The blue module, the most correlated module in PPvNN, was enriched for lipid metabolic pathways as well as biosynthetic pathways. This finding is in line with the results of Gudjonsson et al. [3, 24] that uncovered DE genes also enriched for lipid metabolism along with biosynthetic pathways. Several of the downregulated genes enriching for these pathways in Gudjonsson et al. [24] were also downregulated in our analysis and found in the blue module, including *PPARA*, *ELOVL3*, *ACSBG1*, and *HSD3B1*. Gudjonsson et al. [3] inferred that this enrichment for lipid and fatty acid metabolic pathways is associated with defects in the epidermal lipid barrier of psoriatic skin. However, there is mounting evidence that perturbations of lipid metabolic pathways may also be associated with T cell fate and function [25], particularly in T_reg_ cells [26]. It has very recently been shown that short-chain fatty acids promote differentiation of naïve T cells into T_reg_ cells [27, 28] and that dysfunctional T_reg_ cells residing in the skin are thought to contribute to the pathogenesis of psoriasis [29]. Therefore, we found it very interesting that genes involved in short-chain fatty acid metabolic pathways including propionate metabolism and butyrate metabolism were enriched in the blue module and that the majority of these genes were significantly downregulated, which could potentially lead to T_reg_ dysfunction. As recent studies of immune cell metabolism in other autoimmune diseases such as rheumatoid arthritis and systemic lupus erythematosus suggest that disease and T cell specific metabolic profiles regulate pathogenic responses [25], our data suggest that future T cell metabolism studies in psoriasis are warranted.

### Module enriched for olfactory receptor activity

One of our most surprising and intriguing findings was the enrichment of negatively regulated olfactory receptor genes in the PPvNN salmon module (second most correlated module in PPvNN). Since the discovery that olfactory receptors are expressed in non-nasal tissue, olfactory receptor expression has been observed in skin tissue and specifically in keratinocytes, dendritic cells, and melanocytes. Most recently, Busse et al.[30] discovered that *OR2AT4*, an olfactory receptor gene, is expressed in keratinocytes and that exposure to a synthetic odorant activated a calcium signal transduction pathway that induced wound healing. Jabbari et al. [31] and Li et al. [5] revealed that *OR2T10, OR2T11, OR52B6, OR9Q1, OR10V1, OR1L8, OR2A1, OR2A20P, OR2A42,* and *OR2A9P* were down-regulated while *OR1J1* and *ORMDL2* were up-regulated*.* While *OR2AT4* is not a DEG in our analysis or in previous studies [5, 31, 32] and is not a member of a module significantly correlated with psoriasis in PPvNN or PPvPT, we nonetheless observed that the salmon module was significantly enriched for olfactory signaling and transduction canonical pathways, a finding that bolsters our previous analysis of this data using a complementary coexpression analysis method [21]. Furthermore, Li et al. [5] had also reported that a module detected via WGCNA that was significantly correlated with psoriasis was significantly enriched for “olfactory receptor activity”.

### Hub genes in blue module

Within the blue module, we identified 33 hub genes or genes with high GS (GS ≥ 10) and high connectivity between genes (MM ≥ 0.8) (Fig. 2b) of which 32 were DEGs, including 5 lncRNAs (Additional file 5). The gene with the highest GS was *HOXA10* while the gene with the highest MM was *GGH. HOXA10*, is a homeobox gene that encodes a DNA-binding transcription factor that has been implicated in endometriosis [33], oncogenesis [34], and most recently in innate immune response regulation [35]. In a study of B cell differentiation, Yasmeen et al. [36] demonstrated that the knockout of an aldehyde dehydrogenase-1 enzyme involved in retinol metabolism and retinoic acid synthesis and encoded by the ALDH1 family of genes resulted in increased expression of *HOXA10* by downregulating the expression of the anti-inflammatory transcription factor peroxisome proliferator-activated receptor *PPARG. ALDH1L1*, another hub gene in the blue module, is negatively coexpressed with *HOXA10* and while *PPARG* is not a hub gene in the blue module, it is significantly downregulated in PPvNN. Hub gene *CD200*, is a gene that encodes for a transmembrane glycoprotein and has been shown to attenuate inflammatory response and promote immune tolerance [37, 38] and is downregulated in PPvNN. The gene encoding for fatty acid binding protein, *FABP5*, is another hub gene was upregulated and highly connected in the PPvNN blue module and has been shown previously to interact with psoriasin (*S100A7*) [39] and is highly expressed in psoriatic epidermal tissue [40]. The final hub gene of note was the transmembrane protein encoding gene, *TMEM57*, which was upregulated and highly connected in PPvNN and while it has not yet been implicated in psoriasis pathogenesis directly, a *TMEM57* variant was found to be assoicated with a biomarker for inflammation in a Sardinian population [41].

### Treatment with adalimumab normalizes perturbed pathways

We found that for a number of GO term enrichments and Broad MSigDB canonical pathways, dysregulated pathways in psoriasis (either overexpressed or underexpressed in PPvNN) reverted towards the baseline after adalimumab treatment in PPvPT (Additional file 2). For instance, while the GO terms, “viral reproductive process”, “nuclear division”, and “mitosis” are significantly enriched for upregulated genes in PPvNN, these same terms are significantly enriched for downregulated genes in PPvPT. The top canonical pathways in PPvNN, “olfactory transduction”, “genes involved in olfactory signaling pathway”, and “genes involved in cell cycle”, also reversed direction, suggesting that these return towards a pre-psoriatic baseline with biologic treatment. To investigate the possibility that treatment with adalimumab may have caused reversal of pathway direction beyond the baseline in controls, we also examined the enrichment for GO terms and canonical pathways in NN vs PT. We found that for all of the pathways that reverted towards the basline in PPvPT, none appear to “overshoot” the baseline.

### Evaluation of putative psoriasis autoantigen

While it has long been thought that an autoantigen may trigger T-cell activation and subsequent development of psoriasis in susceptible individuals, characterization of the responsible autoantigen has been elusive. Very recently, Lande et al. [42] revealed that LL37/CAMP is recognized as an autoantigen by T cells in nearly 50 % of psoriasis patients and much more frequently in cases of moderate-to-severe psoriasis. *CAMP* was marginally downregulated in PPvNN (*p* = 0.01, q = 0.059) and was coexpressed in the blue module in PPvNN. *CAMP* was also significantly downregulated in PPvPT (*p* = 1.5e-3, q = 1.4e-2) but was not coexpressed in any module. Genes that were highly coexpressed with *CAMP* within the blue module were enriched for metabolic pathways such as fatty acid metabolic process and lipid biosynthesis, pathways that were enriched for in the blue module as a whole.

## Conclusions

In summary, combining complementary systems biology approaches such as WGCNA with DE analysis has significant advantages over DE analysis alone. For instance, while single gene DE analysis revealed the downregulation of lipid biosynthesis and fatty acid metabolism, network analysis revealed specific short-chain fatty acid metabolic pathways and how these genes may be interacting with each other. We also found that olfactory receptor signaling is significantly enriched for in one of the top associated modules in PPvNN, an interesting observation in light of recent discoveries highlighting the role of olfactory receptors in signal transduction and wound healing in the skin. We discovered that the majority of the top significantly associated modules were composed of lncRNAs, with 90 % of the top 10 PPvNN modules consisting of at least 80 % lncRNAs and 70 % of the top 10 PPvPT modules consisting of at least 70 % lncRNAs. This suggests that lncRNAs likely play a significant role in the regulation of critical pathways in the pathogenesis of psoriasis. Here for the first time we have also described the impact of the TNF-α inhibitor, adalimumab, on these gene networks, with dysregulated pathways reverting back to a pre-psoriatic baseline.

We believe that future studies of populations of isolated individual cell types (i.e. keratinocytes, T cell subsets, dendritic cells, etc.) and single-cell approaches will allow researchers to precisely match each gene network to a particular cell type, shedding further light on which cells are triggering psoriasis and which cells may be conferring resistance to currently available therapies. This matching of gene networks to cell type (and sub cell type) may also aid in functional analyses of lncRNAs, a vast majority of which have no known function. These functional analyses will likely involve the use of siRNA or the more recently developed CRISPRi technologies to perturb genes of interest in each network.

## Additional files

Additional file 1: List of differentially expressed genes. The full list of genes that were differentially expressed (FDR ≤ 0.05) in (1) PPvNN and (2) PPvPT. (XLS 709 kb)
Additional file 2: List of GO terms enriched for amongst differentially expressed genes. The full list of GO terms that are enriched for amongst the differentially expressed genes in (1) PPvNN and (2) PPvPT. Direction indicates whether genes enriching for a given GO term are overexpressed (up) or underexpressed (down). (XLS 336 kb)
Additional file 3: List of GO and Broad MSigDB terms enriched for in the top correlated modules of PPvNN. The full list of GO and Broad MSigDB terms enriched for (p ≤ 0.005) in the blue and salmon modules of PPvNN. (XLS 237 kb)
Additional file 4: List of GO and Broad MSigDB terms enriched for in the top correlated modules of PPvPT. The full list of GO and Broad MSigDB terms enriched for (p ≤ 0.005) in the sienna3 and lightyellow modules of PPvPT. (XLS 83 kb)
Additional file 5: Hub genes of the blue module. List of the hub genes in the PPvNN blue module (GS ≥ 10, MM ≥ 0.8). (TXT 747 bytes)
Additional file 6: Intramodular analysis of most correlated modules. Plots of MM vs GS to explore hub genes for the top 3 most correlated modules in (1) PPvNN and (2) PPvPT. (PDF 730 kb)

## Abbreviations

CRISPRi: Clustered regularly interspaced short palindromic repeats interference
DEG: Differentially expressed gene
FDR: False-discovery rate
GO: Gene ontology
GS: Gene significance
KEGG: Kyoto Encyclopedia of Genes and Genomes
lincRNA: Long intergenic non-coding RNA
lncRNA: Long non-coding RNA
ME: Module eigengene
MM: Module membership
NN: Healthy control patient
PP: Psoriasis patient
PT: Psoriasis patient, post-treatment
qPCR: quantitative polymerase chain reaction
siRNA: Small-interfering RNA
TNF-α: Tumor necrosis factor alpha
TOM: Topological overlap matrix
WGCNA: Weighted gene coexpression network analysis

## References

[1] Bowcock AM, Shannon W, Du F, Duncan J, Cao K, Aftergut K, Catier J, Fernandez-Vina MA, Menter A (2001). Insights into psoriasis and other inflammatory diseases from large-scale gene expression studies. Hum Mol Genet.

[2] Zhou X, Krueger JGJG, Kao M-CJMC, Lee E, Du F, Menter A, Wong WHWH, Bowcock AMAM (2003). Novel mechanisms of T-cell and dendritic cell activation revealed by profiling of psoriasis on the 63,100-element oligonucleotide array. Physiol Genomics.

[3] Gudjonsson JE, Ding J, Johnston A, Tejasvi T, Guzman AM, Nair RP, Voorhees JJ, Abecasis GR, Elder JT (2010). Assessment of the psoriatic transcriptome in a large sample: additional regulated genes and comparisons with in vitro models. J Invest Dermatol.

[4] Elder JT, Bruce AT, Gudjonsson JE, Johnston A, Stuart PE, Tejasvi T, Voorhees JJ, Abecasis GR, Nair RP (2010). Molecular dissection of psoriasis: integrating genetics and biology. J Invest Dermatol.

[5] Li B, Tsoi LC, Swindell WR, Gudjonsson JE, Tejasvi T, Johnston A, Ding J, Stuart PE, Xing X, Kochkodan JJ, Voorhees JJ, Kang HM, Nair RP, Abecasis GR, Elder JT (2014). Transcriptome Analysis of Psoriasis in a Large Case–control Sample: RNA-Seq Provides Insights into Disease Mechanisms. J Invest Dermatol.

[6] Stuart JM (2003). A Gene-Coexpression Network for Global Discovery of Conserved Genetic Modules. Science (80-).

[7] Schäfer J, Strimmer K (2005). An empirical Bayes approach to inferring large-scale gene association networks. Bioinformatics.

[8] Cokus S, Rose S, Haynor D, Grønbech-Jensen N, Pellegrini M (2006). Modelling the network of cell cycle transcription factors in the yeast Saccharomyces cerevisiae. BMC Bioinformatics.

[9] Langfelder P, Horvath S (2008). WGCNA: an R package for weighted correlation network analysis. BMC Bioinformatics.

[10] Subramanian A, Tamayo P, Mootha VK, Mukherjee S, Ebert BL, Gillette MA, Paulovich A, Pomeroy SL, Golub TR, Lander ES, Mesirov JP (2005). Gene set enrichment analysis: a knowledge-based approach for interpreting genome-wide expression profiles. Proc Natl Acad Sci U S A.

[11] Horvath S, Zhang B, Carlson M, Lu KV, Zhu S, Felciano RM, Laurance MF, Zhao W, Qi S, Chen Z, Lee Y, Scheck AC, Liau LM, Wu H, Geschwind DH, Febbo PG, Kornblum HI, Cloughesy TF, Nelson SF, Mischel PS (2006). Analysis of oncogenic signaling networks in glioblastoma identifies ASPM as a molecular target. Proc Natl Acad Sci U S A.

[12] Presson AP, Sobel EM, Papp JC, Suarez CJ, Whistler T, Rajeevan MS, Vernon SD, Horvath S (2008). Integrated Weighted Gene Co-expression Network Analysis with an Application to Chronic Fatigue Syndrome. BMC Syst Biol.

[13] Hu S, Zhou M, Jiang J, Wang J, Elashoff D, Gorr S, Michie SA, Spijkervet FKL, Bootsma H, Kallenberg CGM, Vissink A, Horvath S, Wong DT (2009). Systems biology analysis of Sjögren’s syndrome and mucosa-associated lymphoid tissue lymphoma in parotid glands. Arthritis Rheum.

[14] Huan T, Zhang B, Wang Z, Joehanes R, Zhu J, Johnson AD, Ying S, Munson PJ, Raghavachari N, Wang R, Liu P, Courchesne P, Hwang S-JJ, Assimes TL, McPherson R, Samani NJ, Schunkert H, Meng Q, Suver C, O’Donnell CJ, Derry J, Yang X, Levy D (2013). A systems biology framework identifies molecular underpinnings of coronary heart disease. Arterioscler Thromb Vasc Biol.

[15] Mirza AH, Berthelsen CH, Seemann SE, Pan X, Frederiksen KS, Vilien M, Gorodkin J, Pociot F (2015). Transcriptomic landscape of lncRNAs in inflammatory bowel disease. Genome Med.

[16] Palau N, Julià A, Ferrándiz C, Puig L, Fonseca E, Fernández E, López-Lasanta M, Tortosa R, Marsal S (2013). Genome-wide transcriptional analysis of T cell activation reveals differential gene expression associated with psoriasis. BMC Genomics.

[17] Kim D, Pertea G, Trapnell C, Pimentel H, Kelley R, Salzberg SL (2013). TopHat2: accurate alignment of transcriptomes in the presence of insertions, deletions and gene fusions. Genome Biol.

[18] Pruitt KD, Brown GR, Hiatt SM, Thibaud-Nissen F, Astashyn A, Ermolaeva O, Farrell CM, Hart J, Landrum MJ, McGarvey KM, Murphy MR, O’Leary NA, Pujar S, Rajput B, Rangwala SH, Riddick LD, Shkeda A, Sun H, Tamez P, Tully RE, Wallin C, Webb D, Weber J, Wu W, DiCuccio M, Kitts P, Maglott DR, Murphy TD, Ostell JM (2014). RefSeq: an update on mammalian reference sequences. Nucleic Acids Res.

[19] Hangauer MJ, Vaughn IW, McManus MT (2013). Pervasive transcription of the human genome produces thousands of previously unidentified long intergenic noncoding RNAs. PLoS Genet.

[20] Trapnell C, Hendrickson DG, Sauvageau M, Goff L, Rinn JL, Pachter L (2013). Differential analysis of gene regulation at transcript resolution with RNA-seq. Nat Biotechnol.

[21] Gupta R, Ahn R, Lai K, Mullins E, Debbaneh M, Dimon M, Arron S, Liao W (2015). Landscape of Long Noncoding RNAs in Psoriatic and Healthy Skin. J Invest Dermatol.

[22] Iyer MK (2015). The landscape of long noncoding RNAs in the human transcriptome. Nat Genet.

[23] Fatica A, Bozzoni I (2014). Long non-coding RNAs: new players in cell differentiation and development. Nat Rev Genet.

[24] Gudjonsson JE, Ding J, Li X, Nair RP, Tejasvi T, Qin ZS, Ghosh D, Aphale A, Gumucio DL, Voorhees JJ, Abecasis GR, Elder JT (2009). Global gene expression analysis reveals evidence for decreased lipid biosynthesis and increased innate immunity in uninvolved psoriatic skin. J Invest Dermatol.

[25] Yang Z, Matteson EL, Goronzy JJ, Weyand CM (2015). T-cell metabolism in autoimmune disease. Arthritis Res Ther.

[26] Cipolletta D, Feuerer M, Li A, Kamei N, Lee J, Shoelson SE, Benoist C, Mathis D (2012). PPAR-γ is a major driver of the accumulation and phenotype of adipose tissue Treg cells. Nature.

[27] Arpaia N, Campbell C, Fan X, Dikiy S, van der Veeken J, DeRoos P (2013). Metabolites produced by commensal bacteria promote peripheral regulatory T-cell generation. Nature.

[28] Haghikia A, Jörg S, Duscha A, Berg J, Manzel A, Waschbisch A, Hammer A, Lee D-H, May C, Wilck N, Balogh A, Ostermann AI, Schebb NH, Akkad DA, Grohme DA, Kleinewietfeld M, Kempa S, Thöne J, Demir S, Müller DN, Gold R, Linker RA (2015). Dietary Fatty Acids Directly Impact Central Nervous System Autoimmunity via the Small Intestine. Immunity.

[29] Rodriguez RS, Pauli M, Neuhaus I, Al E (2014). Memory regulatory T cells reside in human skin. J Clin Invest.

[30] Busse D, Kudella P, Grüning N-M, Gisselmann G, Ständer S, Luger T, Jacobsen F, Steinsträßer L, Paus R, Gkogkolou P, Böhm M, Hatt H, Benecke H (2014). A Synthetic Sandalwood Odorant Induces Wound-Healing Processes in Human Keratinocytes via the Olfactory Receptor OR2AT4. J Invest Dermatol.

[31] Jabbari A, Suárez-Fariñas M, Dewell S, Krueger JG (2012). Transcriptional profiling of psoriasis using RNA-seq reveals previously unidentified differentially expressed genes. J Invest Dermatol.

[32] Swindell WR, Xing X, Voorhees JJ, Elder JT, Johnston A, Gudjonsson JE (2014). Integrative RNA-seq and microarray data analysis reveals GC content and gene length biases in the psoriasis transcriptome. Physiol Genomics.

[33] Kallen AN, Haines K, Taylor HS (2014). HOXA10 regulates expression of cytokeratin 15 in endometrial epithelial cytoskeletal remodeling. Reprod Sci.

[34] Bei L, Shah C, Wang H, Huang W, Platanias LC, Eklund EA (2014). Regulation of CDX4 gene transcription by HoxA9, HoxA10, the Mll-Ell oncogene and Shp2 during leukemogenesis. Oncogenesis.

[35] Wang H, Bei L, Shah CA, Hu L, Eklund EA (2015). HoxA10 Terminates Emergency Granulopoiesis by Increasing Expression of Triad1. J Immunol.

[36] Yasmeen R, Meyers JM, Alvarez CE, Thomas JL, Bonnegarde-Bernard A, Alder H, Papenfuss TL, Benson DM, Boyaka PN, Ziouzenkova O (1833). Aldehyde dehydrogenase-1a1 induces oncogene suppressor genes in B cell populations. Biochim Biophys Acta - Mol Cell Res.

[37] Rosenblum MD, Olasz EB, Yancey KB, Woodliff JE, Lazarova Z, Gerber KA, Truitt RL (2004). Expression of CD200 on epithelial cells of the murine hair follicle: a role in tissue-specific immune tolerance?. J Invest Dermatol.

[38] Mukhopadhyay S, Plüddemann A, Hoe JC, Williams KJ, Varin A, Makepeace K, Aknin M-L, Bowdish DME, Smale ST, Barclay AN, Gordon S (2010). Immune Inhibitory Ligand CD200 Induction by TLRs and NLRs Limits Macrophage Activation to Protect the Host from Meningococcal Septicemia. Cell Host Microbe.

[39] Ruse M, Broome A-M, Eckert RL (2003). S100A7 (Psoriasin) Interacts with Epidermal Fatty Acid Binding Protein and Localizes in Focal Adhesion-Like Structures in Cultured Keratinocytes. J Invest Dermatol.

[40] Dallaglio K, Marconi A, Truzzi F, Lotti R, Palazzo E, Petrachi T, Saltari A, Coppini M, Pincelli C (2013). E-FABP induces differentiation in normal human keratinocytes and modulates the differentiation process in psoriatic keratinocytes in vitro. Exp Dermatol.

[41] Naitza S, Porcu E, Steri M, Taub DD, Mulas A, Xiao X, Strait J, Dei M, Lai S, Busonero F, Maschio A, Usala G, Zoledziewska M, Sidore C, Zara I, Pitzalis M, Loi A, Virdis F, Piras R, Deidda F, Whalen MB, Crisponi L, Concas A, Podda C, Uzzau S, Scheet P, Longo DL, Lakatta E, Abecasis GR, Cao A (2012). A Genome-Wide Association Scan on the Levels of Markers of Inflammation in Sardinians Reveals Associations That Underpin Its Complex Regulation. PLoS Genet.

[42] Lande R, Botti E, Jandus C, Dojcinovic D, Fanelli G, Conrad C, Chamilos G, Feldmeyer L, Marinari B, Chon S, Vence L, Riccieri V, Guillaume P, Navarini AA, Romero P, Costanzo A, Piccolella E, Gilliet M, Frasca L (2014). The antimicrobial peptide LL37 is a T-cell autoantigen in psoriasis. Nat Commun.

