# Supplementary material for: Network analysis of psoriasis reveals biological pathways and roles for coding and long non-coding RNAs
Source: BMC Genomics. 2016 Oct 28;17:841. doi: 10.1186/s12864-016-3188-y (PMC5084355; doi:10.1186/s12864-016-3188-y)

Additional File 6

Intramodular analysis using gene significance vs module membership plots

PPvNN

Blue

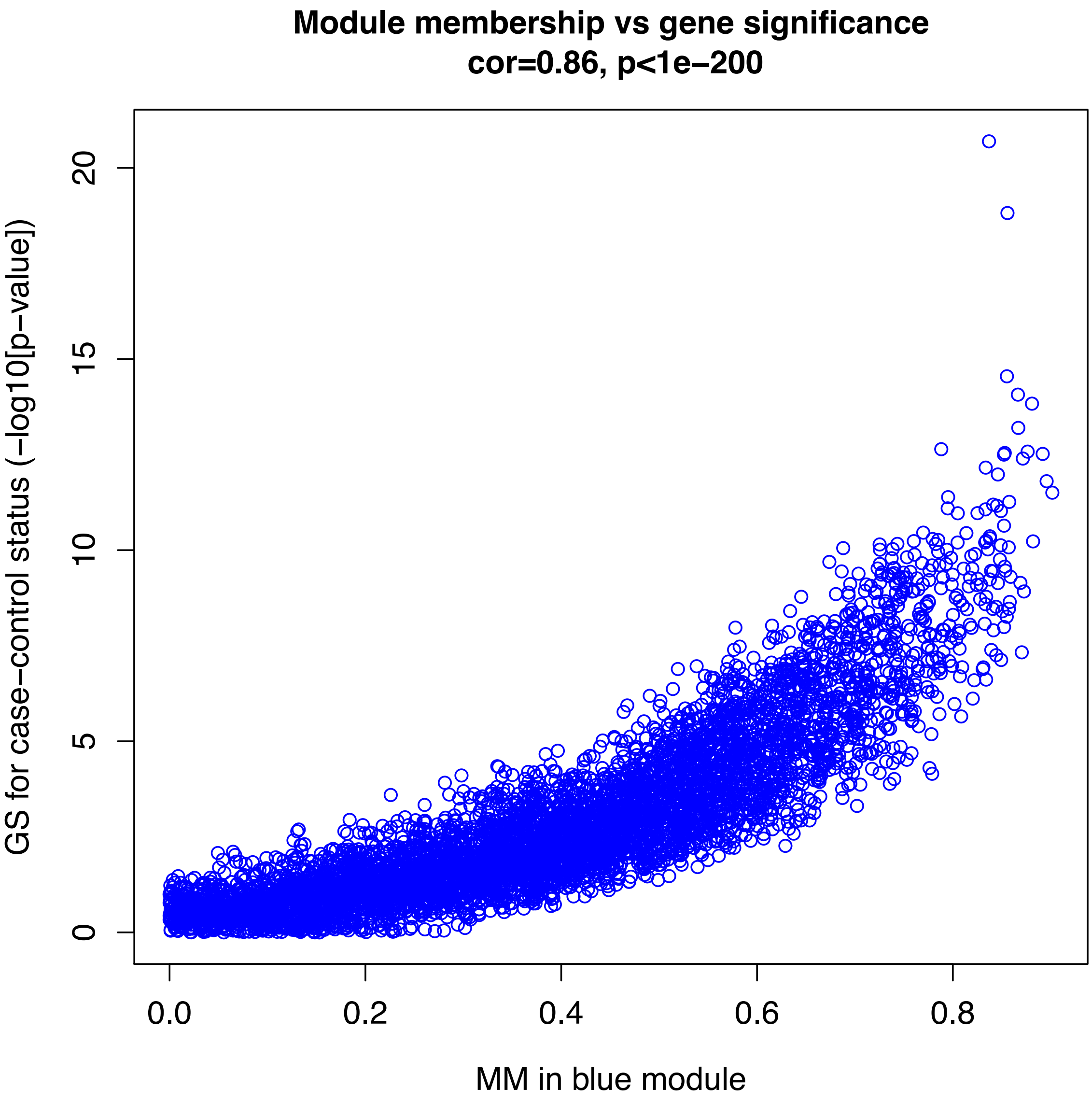

Salmon (PPvNN)

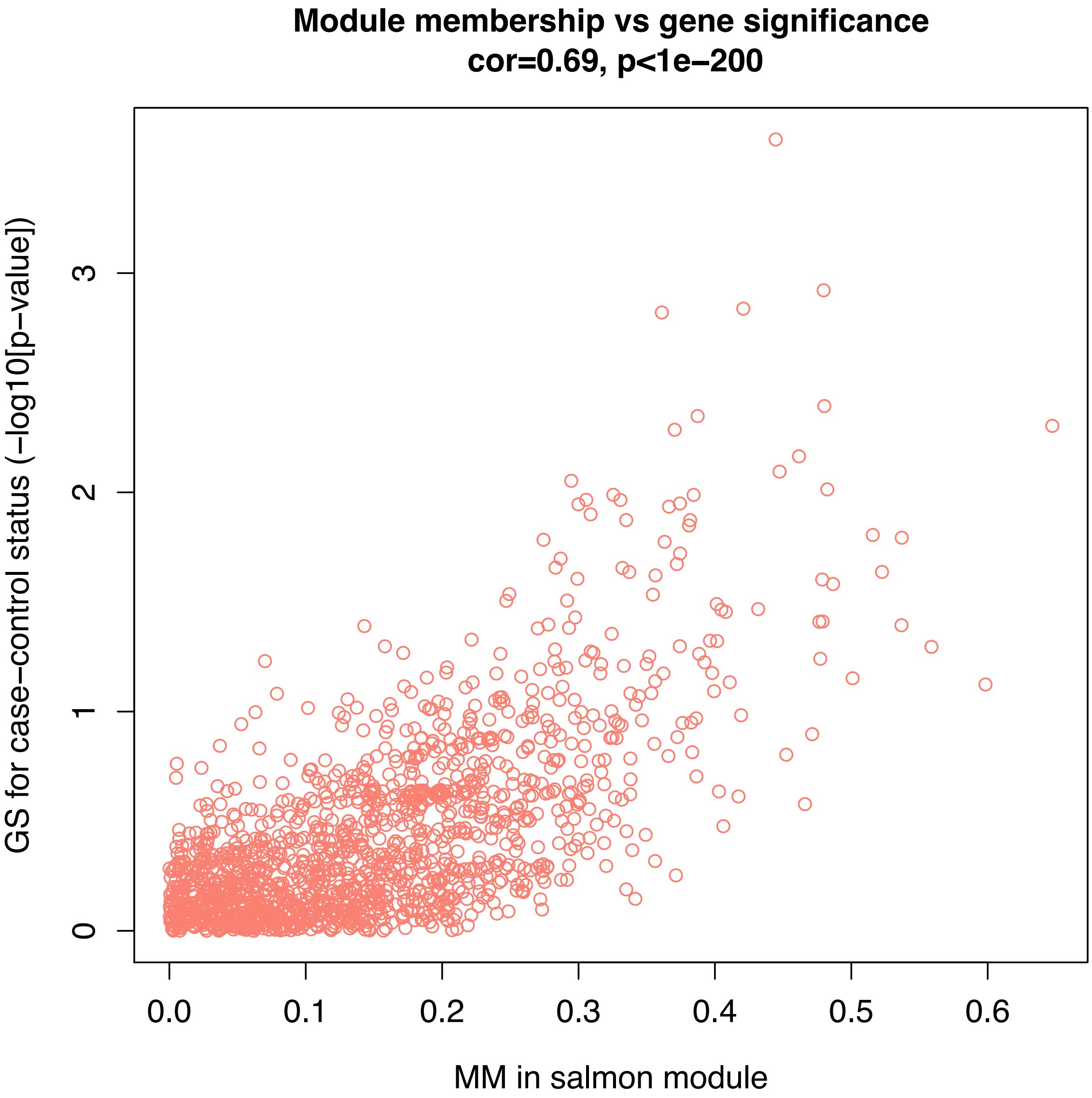

Lavenderblush3

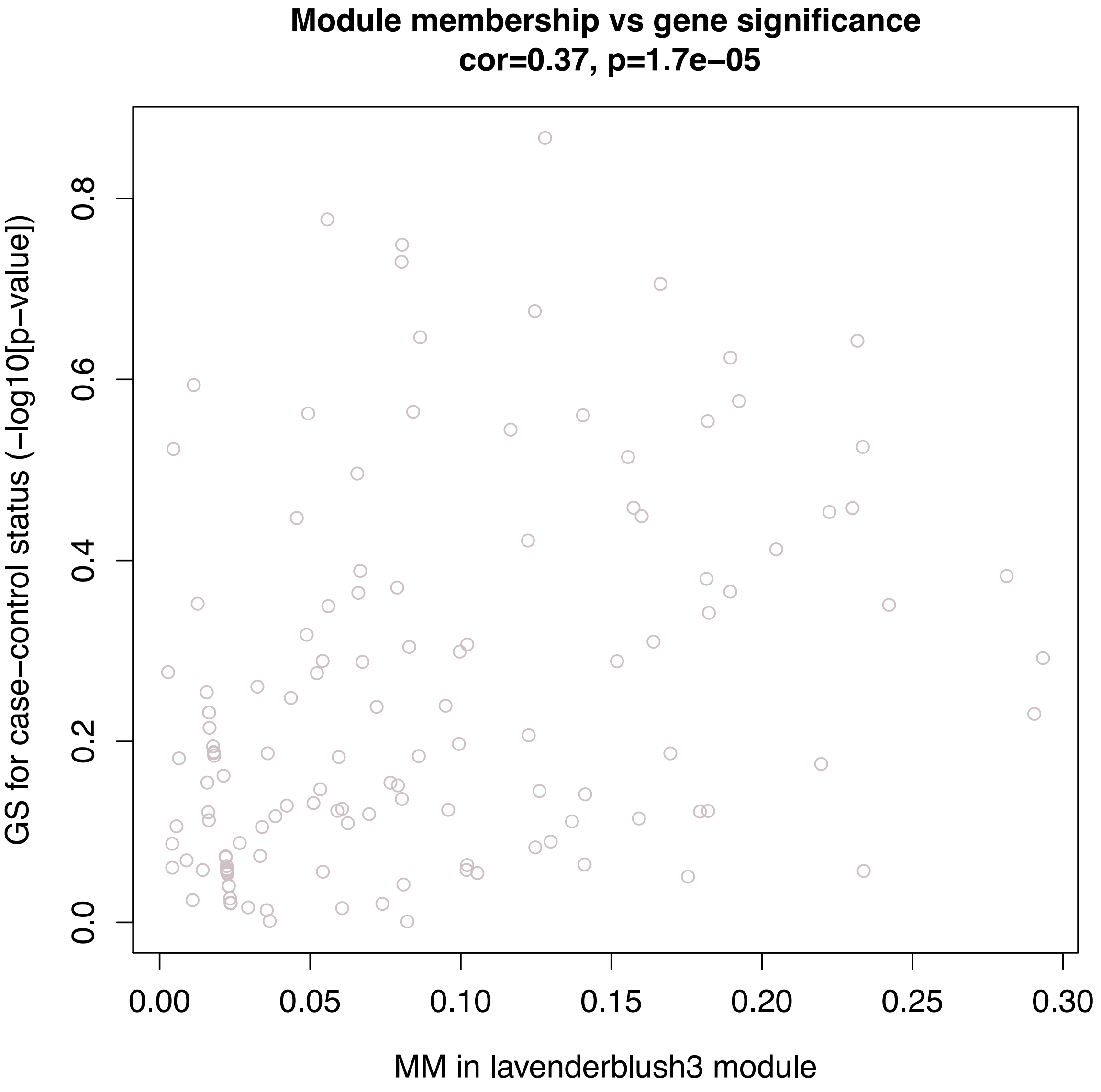

<u>PPvPT</u>

Sienna3

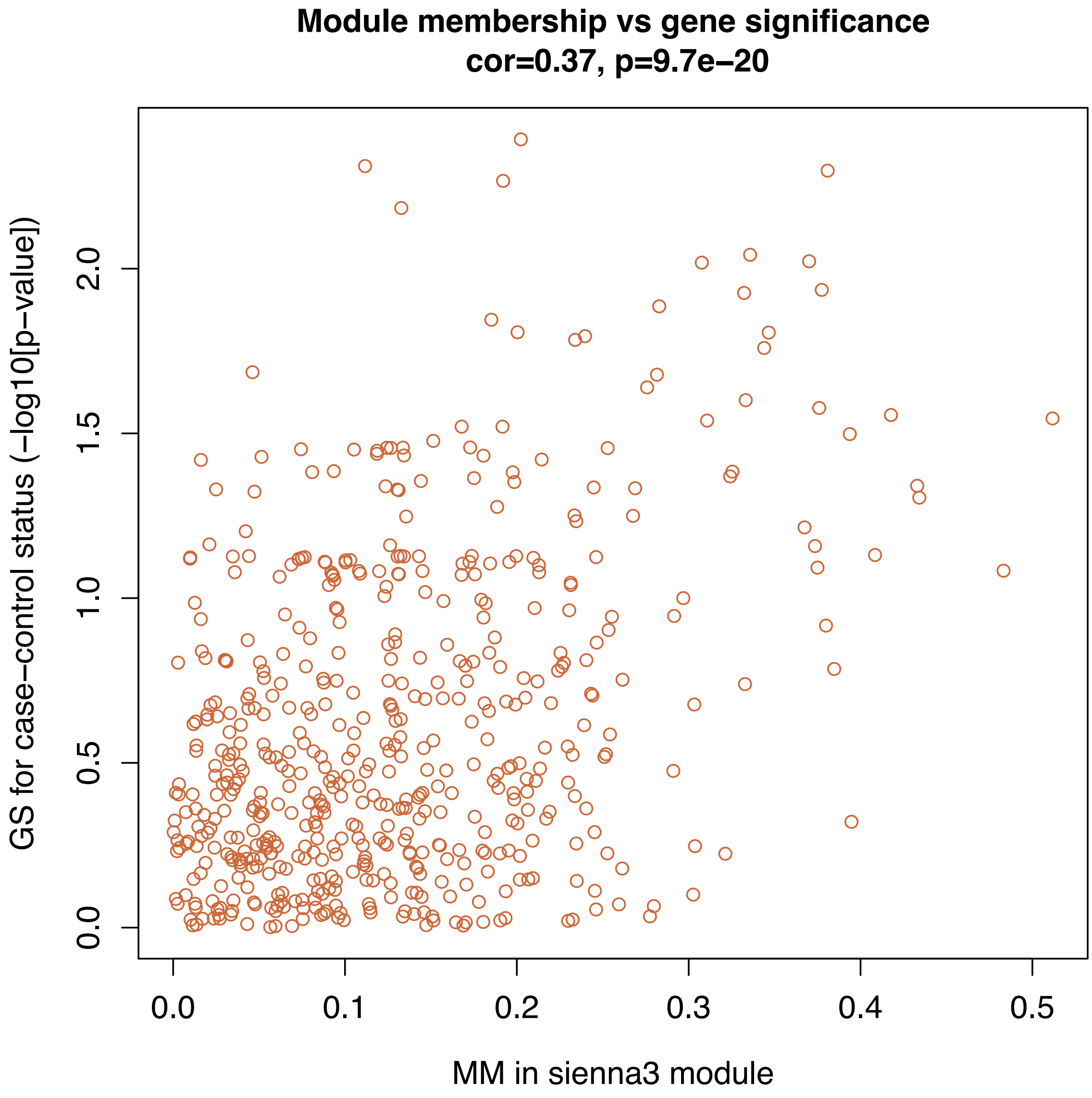

Lightyellow

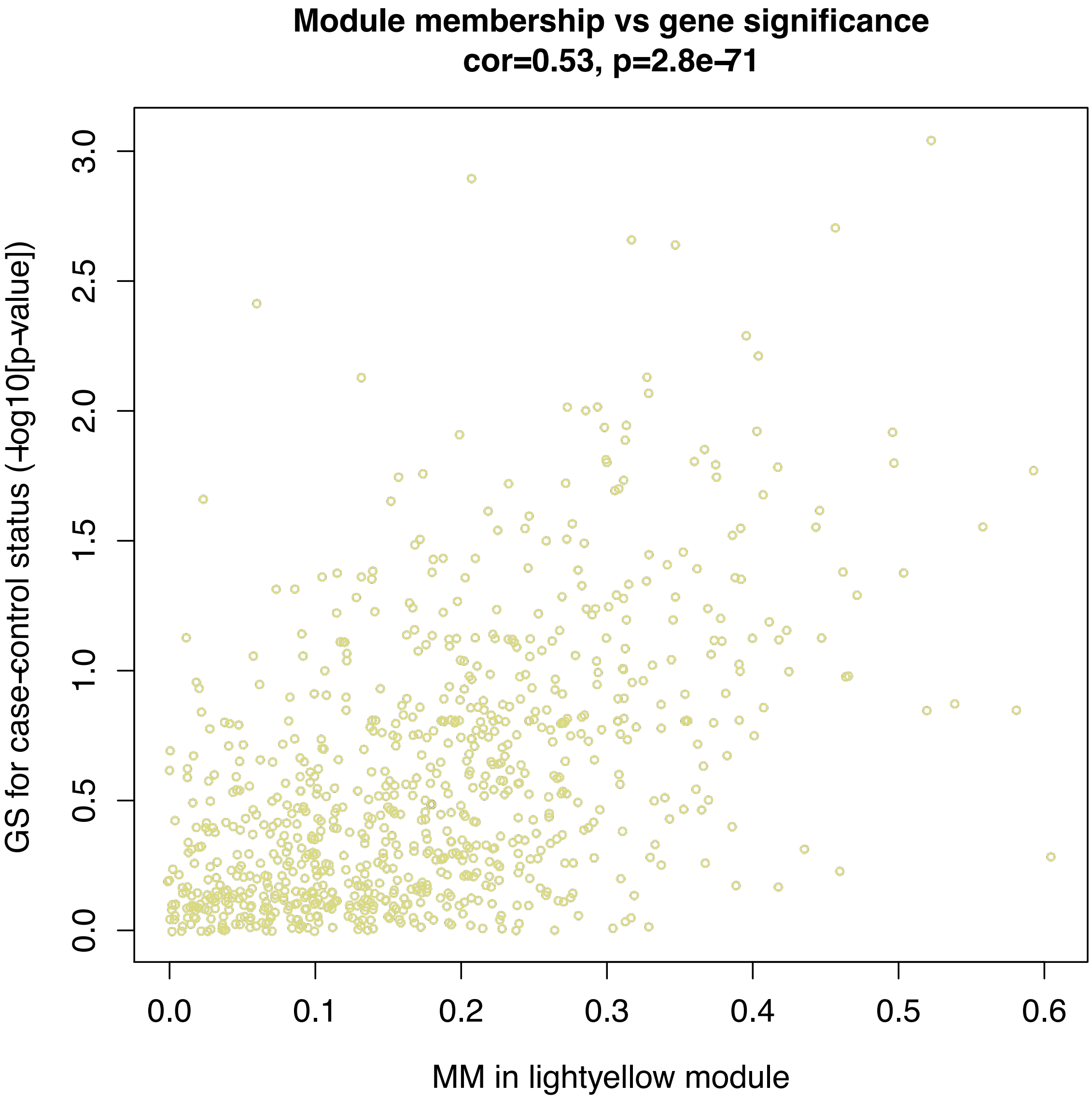

Salmon (PPvPT)

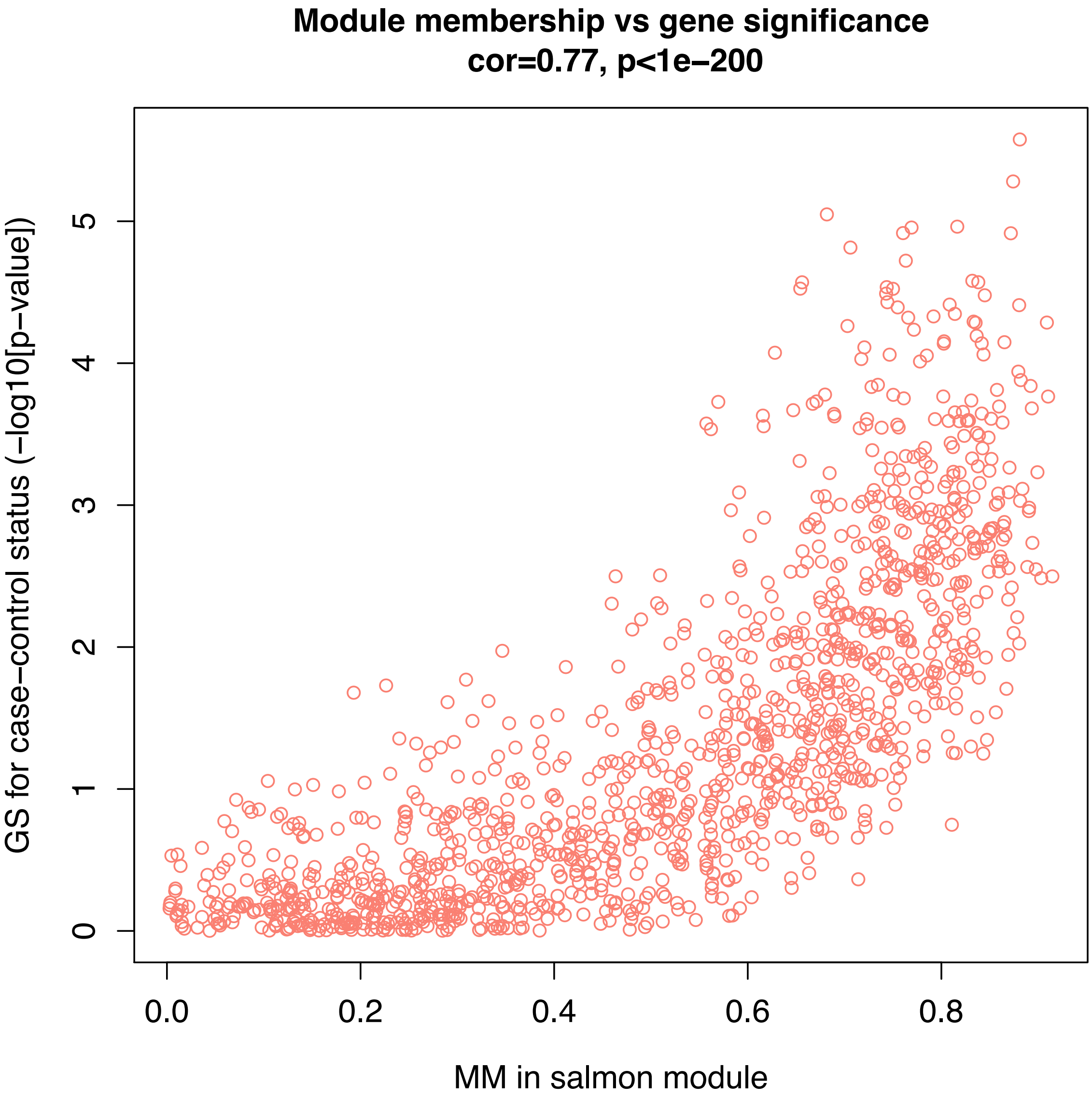

Supplement: Additional file 6: — Intramodular analysis of most correlated modules. Plots of MM vs GS to explore hub genes for the top 3 most correlated modules in (1) PPvNN and (2) PPvPT. (PDF 730 kb) [file 12864_2016_3188_MOESM6_ESM.pdf]
